# EHMTI-0204. Efficacy of SPG stimulation in relieving acute cluster pain: results from >5000 attacks treated during long-term follow-up of the pathway CH-1 study

**DOI:** 10.1186/1129-2377-15-S1-C22

**Published:** 2014-09-18

**Authors:** M Láinez, R Jensen, A May, C Gaul, A Goodman, A Caparso, J Schoenen

**Affiliations:** 1 Neurology, Hospital Clinico Universitario, Valencia, Spain; 2 Neurology, Danish Headache Society, Glostrup, Denmark; 3 Neurology, Universitätsklinikum Hamburg-Eppendorf, Hamburg, Germany; 4 Neurology, University Duisburg-Essen, Essen, Germany; 5 Clinical, Autonomic Technologies Inc., Redwood City, USA; 6 Neurology, CHR de la Citadelle, Liège, Belgium

## Introduction

In the randomized, double-blind, multi-center study of an SPG neurostimulator (Pathway CH-1 study) 68% of patients experienced clinically significant improvements.

## Aim

The aim of this interim analysis is to evaluate acute response of SPG stimulation therapy for the treatment of CCH during Long-Term Follow-Up (LTFU).

## Method

43 patients with refractory CCH (minimum 4 attacks/week) were enrolled in the Pathway CH-1 study; 33 continued into LTFU (from an average of 14 months through up to 3 years following neurostimulator insertion). Each attack treated with SPG stimulation therapy was evaluated for effective therapy (relief from moderate or greater pain, or freedom from mild pain). All evaluable attacks during the LTFU period through March 2014 were included in the analysis.

## Results

Twenty-six patients provided data for treatment of at least one cluster attack during LTFU. Time in LTFU was an average of 365 days (range 117-575). Average number of attacks treated per patient was 197 (range 1-1489). A total of 5132 attacks were treated (22% mild initial pain, 47% moderate, 22% severe, 9% very severe). 65% (N=3354/5132) of these attacks achieved effective therapy (59% of mild attacks, 78% of moderate, 62% of severe, 21% of very severe). Average stimulation duration was 12.9 minutes.

## Conclusion

Two-thirds of the more than 5000 cluster attacks evaluated during the LTFU period are effectively treated with SPG stimulation therapy.

No conflict of interest.

